# Investigation of the Relationship between Body Parameters and mAs Using Non-Contact Two-Dimensional Thickness Measurement in Chest Digital Radiography

**DOI:** 10.3390/s23167169

**Published:** 2023-08-14

**Authors:** Jia-Ru Lin, I-Hao Cheng, Yu-Syuan Liang, Jyun-Jie Li, Jen-Ming Tsai, Min-Tsung Wang, Te-Pao Lin, Su-Lan Huang, Ming-Chung Chou

**Affiliations:** 1 Department of Radiology, Kaohsiung Armed Force General Hospital, Kaohsiung 802, Taiwan; t52565@hotmail.com (J.-R.L.); allyouplayla@hotmail.com (I.-H.C.); jay720920@gmail.com (J.-J.L.); jm6611@gmail.com (J.-M.T.); surgeon.wang63@gmail.com (M.-T.W.); pao699067@gmail.com (T.-P.L.); slhuang2003@gmail.com (S.-L.H.); 2 Department of Radiation Oncology, Kaohsiung Chang Gung Memorial Hospital, Kaohsiung 833, Taiwan; d0w0b123@gmail.com; 3 Department of Medical Imaging and Radiological Sciences, Kaohsiung Medical University, Kaohsiung 807, Taiwan; 4 Department of Medical Research, Kaohsiung Medical University Hospital, Kaohsiung 807, Taiwan; 5 Center for Big Data Research, Kaohsiung Medical University, Kaohsiung 807, Taiwan

**Keywords:** body parameters, chest radiography, mAs, automatic exposure control, non-contact infrared sensor

## Abstract

The current study aimed to investigate the relationship between body parameters and the current–time product (mAs) in chest digital radiography using a non-contact infrared thickness-measurement sensor. An anthropomorphic chest phantom was first used to understand variations in mAs over multiple positionings during chest radiography when using the automatic exposure control (AEC) technique. In a human study, 929 consecutive male subjects who underwent regular chest examinations were enrolled, and their height (H), weight (W), and body mass index (BMI) were recorded. In addition, their chest thickness (T) was measured at exhalation using a non-contact infrared sensor, and chest radiography was then performed using the AEC technique. Finally, the relationship between four body parameters (T, BMI, T*BMI, and W/H) and mAs was investigated by fitting the body parameters to mAs using three curve models. The phantom study showed that the maximum mAs was 1.76 times higher than the lowest mAs during multiple positionings in chest radiography. In the human study, all chest radiographs passed the routine quality control procedure and had an exposure index between 100 and 212. In curve fitting, the comparisons showed that W/H had a closer relationship with mAs than the other body parameters, while the first-order power model with W/H fitted to mAs performed the best and had an R-square of 0.9971. We concluded that the relationship between W/H and mAs in the first-order power model may be helpful in predicting the optimal mAs and reducing the radiation dose for chest radiography when using the AEC technique.

## 1. Introduction

During chest radiography, exposure parameters, such as kVp and mAs, are usually adjusted according to the patient’s body parameters and are intimately associated with image quality [1,2,3,4,5]. In clinical practice, exposure charts are usually required to obtain clinical radiographs of adequate image quality without over- or underexposure by considering the specifications of body part, thickness, and image receptor before the radiography is performed [6,7]. However, body thickness measurements, which were previously determined using a caliper/ruler [2], are prone to errors, with inaccurate thickness measurements potentially leading to improper exposure and inadequate image quality. In line with this, X-ray images of inadequate quality must be re-acquired, and the radiation dose to the patient must be increased [8,9,10]. Therefore, images of patients whose body parameters could not be accurately measured may have to be acquired with slightly higher exposure parameters to increase image quality.

Although automatic exposure control (AEC) has been widely used in radiography to avoid overexposure with high accuracy [11,12,13,14] and has the potential to improve the radiographic image quality [15], the ion chambers or solid-state sensors placed behind the flat-panel detectors (FPDs) may likely under- or overestimate the radiation dose due to incorrect set-up conditions or improper positioning [7,16,17]. Ideally, when the detected exposure reaches a predefined threshold (maximum mAs), the AEC system will automatically terminate the tube current to avoid overexposure. However, if the patient is positioned such that their dense/thick body part, bones, or metal implants are immediately in front of the ion chambers, fewer X-ray photons would be expected to penetrate the body part and reach the AEC sensor, adversely leading to patient overexposure [7,17]. Conversely, when the patient is positioned such that their thin or air-filled body portions are immediately in front of the AEC sensor, more X-ray photons would penetrate the body portions and reach the sensor and may consequently cause underexposure to patients. It has been shown that image quality is intimately correlated with radiation dose, and higher exposure may lead to better radiographic quality [18,19]. Although overexposure may provide superb signal-to-noise ratio in radiographs, it potentially causes signal saturation and loss of information in the thin body portions or soft tissue areas, and the saturated images are not suitable for diagnosis [17,19,20]. In contrast, underexposure is due to insufficient X-ray photons generated for imaging, so the underexposed images may exhibit more quantum noise/mottle in thicker portions of the anatomy [6,19,20]. Therefore, when performing radiography with the AEC technique, appropriate radiological settings and patient positioning are important to maintain image quality and reduce unnecessary radiation exposure to patients [21].

Some previous studies have suggested that body parameters such as thickness, weight, and height are useful in properly adjusting the exposure parameters [1,2,3,4], in compliance with the “as low as reasonably achievable” (ALARA) principle [22], but it remains unclear which body parameter exhibits the closer relationship with mAs during chest radiography. As mentioned previously, body thickness measurements obtained using a ruler are prone to errors and may result in improper exposure parameters and an unstable image quality. Such a manual thickness measurement requires physical contact with patients, which increases the risk of virus infection during radiography [23,24,25]. Moreover, although radiographic examinations are usually performed on a two-dimensional field-of-view (FOV), ruler measurements cannot accurately obtain the average thickness of the body part to be examined.

Therefore, there is an urgent clinical need to develop a fast, accurate, and non-contact sensor for measuring two-dimensional body thickness as an adjunct to the AEC technique in order to properly predict the exposure parameters and reduce radiation overdose for patients undergoing radiography. As such, the purposes of the current study were three-fold: (1) to utilize a non-contact infrared sensor to accurately measure the average body thickness; (2) to investigate the relationship between body parameters and the current–time product (mAs); (3) to identify the best prediction model by comparing the goodness-of-fit between four body parameters and three models of chest radiography when using the AEC technique.

## 2. Materials and Methods

### 2.1. Non-Contact Infrared Sensor

This study employed a Kinect^®^ (Microsoft, Redmond, WA, USA) device with a built-in infrared sensor for the two-dimensional measurement of body thickness. The sensor is capable of generating a two-dimensional distance map based on the reflection of infrared rays traveling between the device and an object [26,27,28]. Before utilizing the device, this study initially performed non-contact distance measurements for five different distances between 100 and 180 cm to assess the linearity of the distance-response function of the Kinect sensor.

**Figure 1:**
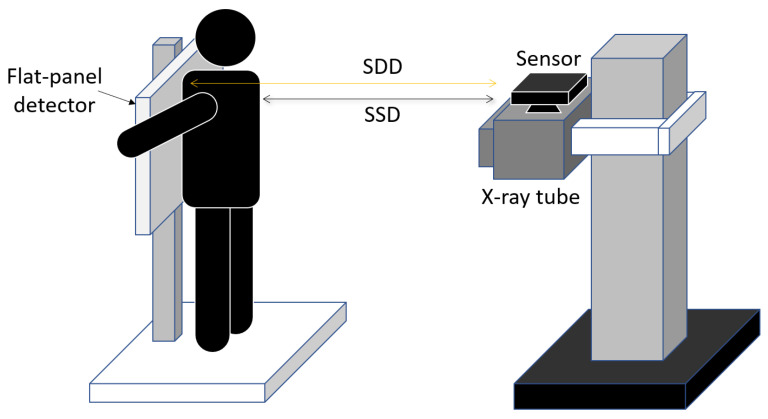
Measurement of body thickness using the non-contact sensor. The sensor was immobilized on top of the X-ray tube for two-dimensional thickness measurement. The average thickness was obtained by subtracting the sensor-to-surface distance (SSD) from the sensor-to-detector distance (SDD) or the baseline distance.

Afterwards, the distance-response function of the device between the actual and the measured distance was utilized for calibration. In the human study, the device was installed on the head of the X-ray tube, and the average distance between the device and the FPD was measured as a baseline. Before chest radiography, a subject was positioned by leaning closely on the FPD, after which the average distance between the sensor and the body surface (SSD) was measured at exhalation phase via a non-contact approach. Finally, the SSD was subtracted from the baseline to obtain the average body part thickness, as illustrated in Figure 1.

### 2.2. Phantom Study

**Figure 2:**
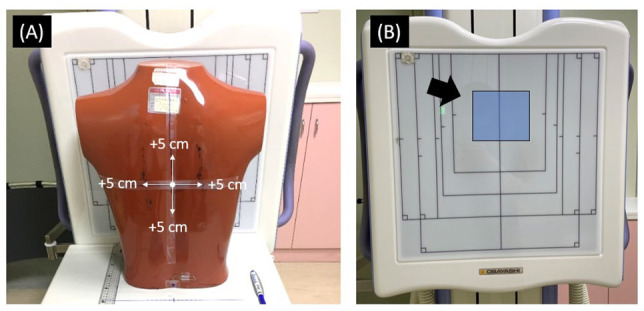
The anthropomorphic chest phantom used in this study. (**A**) The phantom was placed at the central FOV and then displaced by 1, 2, 3, 4, and 5 cm toward the right, left, superior, and inferior directions for multiple image acquisitions. (**B**) A solid-state AEC sensor was placed behind the FPD at the upper center area as indicated by the black arrow.

To understand the extent of overexposure caused by positioning variations when using the AEC technique, an anthropomorphic chest phantom was used to acquire multiple X-ray images at different locations at 105 kVp, 200 mA, and a source-to-image distance (SID) of 180 cm. The AEC technique was utilized to automatically terminate the tube current, and the exposure duration was used to obtain the mAs. The phantom was initially positioned at the center FOV and then displaced by 1, 2, 3, 4, and 5 cm toward the right, left, superior, and inferior directions, as shown in Figure 2A. For each location, five repeated X-ray image acquisitions were performed in a digital radiography system (CXDI-401C CANON; FPD-TFT; KXO-32S TOSHIBA), and the resulting mAs and exposure index (EI) were recorded for analysis.

### 2.3. Human Subjects

This prospective study was approved by a local institutional review board (protocol code: 106-028 and date of approval: 11 June 2017). A total of 929 consecutive male subjects (aged 20–85 years) who underwent regular chest screening examinations were enrolled. The inclusion criterion was male subjects with age ≥ 20 years old. The exclusion criteria were (1) age < 20 years old, (2) patients with excessive forward curvature of the back, (3) those unable to cooperate during an X-ray examination. After obtaining informed consent, the height (H), weight (W), and body mass index (BMI) of each subject were determined using a height-and-mass scale. Subsequently, the average chest thickness (T) was determined by measuring at the exhalation phase using the calibrated non-contact thickness measurement device. The device was connected to a standalone computer and was immobilized on the top of an X-ray tube equipped with a digital radiography system (CXDI-401C CANON; FPD-TFT; KXO-32S TOSHIBA). Standing posterior-to-anterior chest radiography was performed on each subject using routine exposure parameters (105 kVp and 250 mA) and SID of 180 cm. The AEC technique was used to automatically control the exposure duration, and the mAs and exposure index (EI) were recorded for statistical analysis.

To assess the diagnostic performance of the human chest radiographs, 14 chest radiographs were randomly selected for visual grading analysis (VGA) by two independent radiologists (T.P.L. and M.T.W.) with experience of 15 and 22 years, respectively. VGA was performed five times (once/week) based on the modified European quality criteria for chest imaging [29]. The intra-class correlation (ICC) analysis was performed to understand the inter-observer variability, and the Wilcoxon signed-rank test was performed to determine if there was a significant difference between the two observers with respect to the VGA scores.

**Figure 3:**
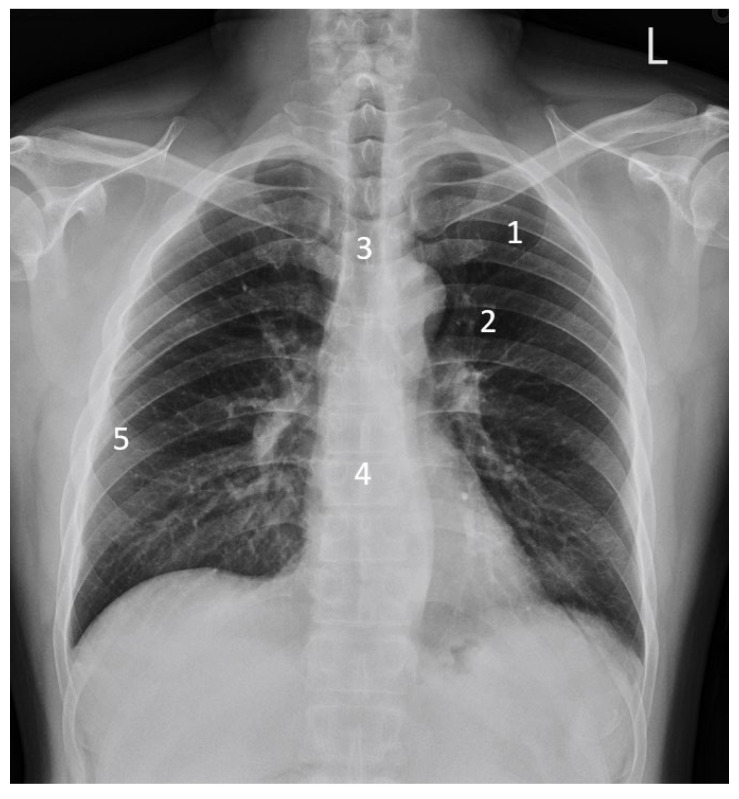
The five body structures assessed using visual grading analysis of chest radiographs. The image was acquired from a 42-year-old male subject with body thickness of 22.03 cm, BMI of 27.78 kg/m^2^, and EI of 160. The subject had a mean VGA score of 3.6 and 4.0 by the two raters. (1) The vessels 3 cm away from the pleural margin. (2) Vessels in the central region. (3) The carina with the main bronchi. (4) The thoracic vertebrae behind the heart. (5) The pleural margin.

During VGA analysis, a 4-point Likert scale (1: not visible, 2: poorly reproduced, 3: adequately reproduced, 4: very well reproduced) was used to evaluate image quality, as shown in Figure 3. The mean VGA scores were calculated, with scores higher than 3 indicating adequate to very good image quality.

### 2.4. Data Analysis

To understand the relationships between the four body parameters (T, BMI, BMI*T, and W/H) and mAs, three models, namely the first-order exponential ($y=a\cdot e^{b\;x}$), second-order polynomial ($y=ax^{2}+bx+c$), and first-order power ($y=ax^{b}+c$) functions, were used for comparisons because they have been previously utilized to investigate the relationship between thickness and radiation dose [1,30]. Accordingly, “*x*” and “*y*” indicate the body parameter and mAs, respectively, whereas “*a*”, “*b*”, and “*c*” indicate the unknown model parameters to be fitted. In this study, curve fitting was performed using the curve-fitting toolbox of MATLAB (Mathworks, Natick, MA, USA). To understand how the curve fits a set of data, the goodness-of-fit test (R^2^) and root-mean-squared errors (RMSE) were compared.

## 3. Results

Results from the non-contact infrared distance measurement device showed an excellent linear relationship between the measured distance and the actual distance, with a slope very close to identity (r = 1.017).

**Figure 4:**
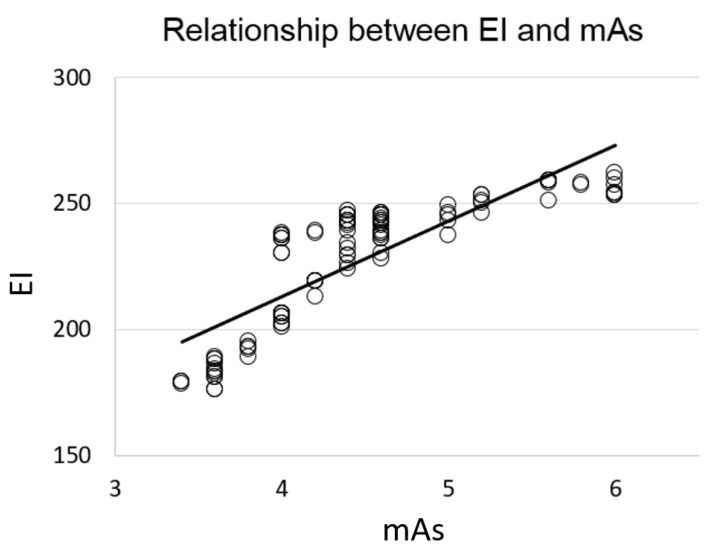
Relationship between the EI and mAs in the phantom chest radiography.

The phantom study showed that the mAs was 4.48 ± 0.71 (mean ± standard deviation), while the EI was 227.4 ± 25.4 (mean ± standard deviation), based on 105 multiple image acquisitions at different positioning locations. At the center position, the mean and standard deviation of mAs were 4.6 ± 0.0. By displacing it from the center to the left and right directions, the mAs was decreased to 3.6 ± 0.0 and 3.5 ± 0.1, respectively. However, when displacing the phantom from the center to the superior and inferior directions, the mAs was changed to 6.0 ± 0.0 and 4.0 ± 0.0, respectively. The data demonstrated that the mAs values used were between 3.4 and 6.0 and the EI ranged from 176 to 262 for the same phantom when using the AEC technique during multiple chest radiography. Correlation analysis revealed that the EI was significantly correlated to the mAs (r = 0.84 and *p* < 0.001) in the 105 phantom images, as shown in Figure 4.

The demographic characteristics of the 929 subjects enrolled in the human study are listed in Table 1. The comparison showed that in all three models, W/H had closer relationship with mAs (higher R^2^ and lower RMSE) than the other body parameters (T, BMI, and T*BMI). Table 2 lists the R-square values and RMSEs for the four body parameters using the three models.

**Figure 5:**
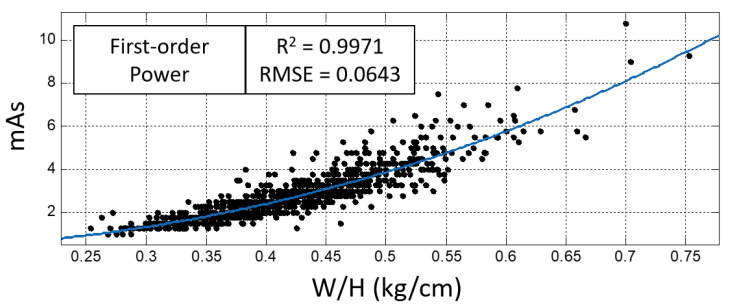
The first-order power model with W/H fitted to mAs ($mAs=17.8{\left(\frac{W}{H}\right)}^{2.26}+0.14$) using all data points exhibited the highest R-square and lowest RMSE.

The comparison further revealed that the first-order power model with W/H fitted to mAs ($mAs=17.8{\left(\frac{W}{H}\right)}^{2.26}+0.14$) had the highest R^2^ = 0.9971 and the lowest RMSE = 0.0643, as shown in Figure 5. The fitted model, which roughly represents the average mAs under the same W/H body parameter, was termed the “50th-percentile” first-order power model.

In VGA analysis, the demographic characteristics of the 14 randomly selected patients are listed in Table 3. The VGA analysis showed that the mean VGA scores of the two raters were consistent and did not differ significantly (mean ± standard deviation = 3.94 ± 0.12 and 3.77 ± 0.22, respectively) across the 14 randomly selected chest radiographs. However, no significant correlation was noted between the mean VGA scores and the EI, and no significant ICC correlation was noted between the two raters (ICC = 0.236, *p* = 0.198).

## 4. Discussion

To the best of our knowledge, this is the first study that has employed a non-contact infrared sensor to measure the average thickness of a body part to be examined using a two-dimensional distance map. The relationship between the body parameters and the exposure parameter mAs was investigated in patients undergoing chest radiography. Notably, our results demonstrated that the non-contact sensor had a linear distance response and was reliable for distance and thickness measurements. The phantom study showed that the AEC technique may lead to an approximately 1.76 times greater overexposure than the lowest mAs during multiple chest radiography with different positioning locations, with both exhibiting clinically acceptable image quality. The results suggest that the use of the AEC technique alone may promote overexposure to patients with identical body parameters in chest radiography. In the human study, the results of curve fitting demonstrated that the four body parameters (T, BMI, T*BMI, and W/H) were closely associated with mAs, with an R-square of >0.95 and an RMSE of <0.3. These findings indicate that those four body parameters are potentially useful when predicting the mAs for chest radiography. Finally, VGA analysis showed that the randomly selected chest radiographs exhibited a mean VGA score of >3.5 points and an EI of >124, but no significant correlation was noted between them.

One previous study collected 180 radiographic examinations to establish a method with an appropriate exposure derived from the EI and thickness [1], but the body thickness was not directly measured from patients. Instead, the chest thickness was only approximated from the W/H ratio using the following equation: chest thickness = $8.47\left(\frac{W}{H}\right)+17.51{\left(\frac{W}{H}\right)}^{0.5}+4.21$ [31]. In addition, their proposed model was a first-order exponential model that considered body thickness, entrance-surface air kerma, and the Bucky factor, which all needed to be determined in advance, so the method could not be implemented if those factors were unknown. Another previous study investigated the relationship between thickness and exposure parameters from 200 radiographic examinations by measuring body thickness manually using a caliper. Although the mAs was positively correlated with body thickness based on the linear regression method [2], the results may be biased due to manual errors in the thickness measurement. However, these two previous studies did not compare body thickness with other body parameters in predicting and correlating with mAs. In contrast, the present study collected 979 radiographic examinations and utilized a non-contact infrared sensor to accurately measure the average chest thickness from a two-dimensional thickness map. Furthermore, the relationships between four body parameters and mAs were investigated using three curve models and the goodness-of-fit was compared to build the best prediction model.

In the human study, the comparisons of the four body parameters demonstrated that W/H exhibited a closer relationship with mAs than the other body parameters (T, BMI, and BMI*T) during digital chest radiography with the AEC technique. Given that the thoracic cavity consists of air-filled lungs, bone, and dense soft tissues, chest thickness alone cannot properly reflect the composition of the chest. In contrast, both W/H and BMI consider body mass and height and had a larger R-square than that of thickness in the three models, suggesting that these two body parameters might better reflect the composition of the chest than body thickness. Although the W/H and BMI were similar in definition, the comparisons showed that W/H outperformed BMI, with a larger R-square value in all three models. Since the W/H can be thought of as the average cross-sectional weight per unit height, the W/H may better reflect the average thickness of total thoracic tissues. As such, W/H has a closer relationship with mAs and may be a more suitable body parameter than the other body parameters for predicting the mAs for patients undergoing chest radiography. Although the chest thickness did not show a closer relationship with mAs than the other body parameters in chest radiography due to the complex composition of the thorax, the thickness may be potentially useful to predict the mAs for other body parts without complex composition, such as the upper and lower extremities. Hence, the body thickness may have a closer relationship with mAs than the other body parameters for the radiography of extremities. Further investigation will be needed to explore the relationship between the thickness and mAs to build the best prediction model of mAs for other body parts.

In the phantom study, the results demonstrated that the AEC technique could generate consistent mAs in repeated chest radiographs at the same position, indicating that AEC is a reliable technique to automatically terminate the tube current during radiography. However, it was noted that the mAs varied from 3.4 to 6.0 mAs when the phantom was displaced toward different directions in chest radiography with the AEC technique. As the radiography was performed on the same chest phantom, it was expected to have identical mAs generated by the AEC technique. Because all the phantom images exhibited adequate image quality, the results indicated that positioning differences may be responsible for the 1.76-fold mAs difference in chest radiography with the AEC technique. According to the ALARA principle, minimally acceptable exposure should be used to acquire a radiograph of adequate image quality, so the 3.4 mAs was likely the minimally acceptable exposure parameter for the chest phantom radiography. Similarly, in the human study, it was also noted that the mAs was different in patients with identical body parameters when using the AEC technique. As all the chest radiographs passed the routine quality control procedure and were accepted for clinical diagnosis, the variations in mAs may be attributable to the positioning differences between those patients with identical body parameters. Specifically, in those patients with a W/H around 0.5, the mAs varied from 2.8 to 6.2 mAs, as shown in Figure 5, implying that positioning variations may lead to a 2.2-fold difference in human chest radiography when using the AEC technique.

Furthermore, the phantom study conducted herein revealed a significant correlation between the EI and mAs in multiple chest radiographs with various positionings, suggesting that higher mAs leads to better image quality in chest radiographs. However, no significant correlation was noted between the EI and the mean VGA scores for human chest radiographs. The EI measures the physical image quality [32,33,34], whereas the VGA score reflects the diagnostic quality of the acquired image [35,36,37]. A physical image quality that is higher than the visually perceptible level suggests an adequate to very good diagnostic image quality during the VGA analysis. Although no significant correlation was noted between the EI and VGA scores, the VGA scores of the randomly selected radiographs were higher than 3 points, indicating that the thoracic structures could be adequately and superbly visualized in the chest radiographs, with an EI between 124 and 196. However, the present study did not perform a VGA analysis for all chest radiographs that had an EI between 100 and 212. Although some chest radiographs had EI values lower than 124, all images passed the routine quality control procedure and were accepted for clinical diagnosis, indicating that the 929 chest radiographs exhibited adequate to very good diagnostic image quality.

The present study found that the best model with the highest R^2^ and lowest RMSE was the first-order power model of W/H. The fitted model obtained from all datasets was able to roughly predict the “50th-percentile” mAs for chest radiography with a 50% reduction in overexposure using the AEC technique. Therefore, the “50th-percentile” model might be used as an adjunct to the AEC technique; together, these may help to minimize the radiation dose to patients while maintaining image quality, in compliance with the ALARA principle in digital radiography. Moreover, the prediction model may be potentially useful for portable radiography units when the AEC technique is not available.

We acknowledge some limitations in this study. First, given that only male subjects were enrolled herein, the relationship between body parameters and mAs in female subjects remains unknown. Because body composition is slightly different between male and female subjects, the best prediction model may be different for male and female subjects. In addition, patients with hunchback were not included, so the applicability of our proposed model to those patients remains unclear and needs further investigation. Second, this study only compared three commonly used curve models. Although these three models were relatively simple, their R^2^ values were higher than 0.95 across the four body parameters. Third, chest radiography was only performed using one digital radiography system of a hospital. To implement our proposed method with a different X-ray unit, it will be necessary to collect a dataset from the new unit and perform the curve fitting to build the best model for predicting the mAs. An extended survey will be needed in the future. Finally, this study did not investigate the relationship between the body parameters and kVp, given that the voltage was usually kept constant for chest radiography. However, further investigation will be needed to understand the relationship between body parameters, mAs, and kVp for patients with extreme obesity.

## 5. Conclusions

In conclusion, the present study utilized a non-contact infrared sensor to estimate two-dimensional body thickness and determined the relationship between four body parameters and mAs during chest radiography. Our results demonstrated that positioning variations could lead to overexposure in chest phantom radiography with the AEC technique, and that W/H was a more suitable body parameter for predicting mAs compared with thickness and BMI when using the first-order power function for human chest radiography. Therefore, we concluded that the relationship between W/H and mAs in the first-order power model may help predict the optimal mAs and reduce the radiation dose for patients undergoing chest radiography when using the AEC technique.

## Figures and Tables

**Table 1:** Demographic characteristics of the 929 male subjects. Data are expressed as mean ± standard deviation.

Age (Years)	Height (cm)	Weight (kg)	BMI (kg/m^2^)	Thickness (cm)	Exposure Index
25.3 ± 7.0	172.7 ± 5.9	72.2 ± 13.2	24.2 ± 4.1	19.1 ± 3.6	145.4 ± 18.1

**Table 2:** Comparison of the R-square and RMSE values according to the four body parameters in the three models, where *a*, *b*, and *c* are the unknown model parameters to be fitted.

Model	Body Parameter	R-Square	RMSE
First-order exponential $f\left(x\right)=a\times e^{b\;x}$	T	0.9936	0.0949
	BMI	0.9932	0.0979
	T*BMI	0.9520	0.2598
	W/H	0.9943	0.0897
Second-order polynomial $f\left(x\right)=ax^{2}+bx+c$	T	0.9888	0.1258
	BMI	0.9957	0.0778
	T*BMI	0.9942	0.0901
	W/H	0.9961	0.0736
First-order power $f\left(x\right)=ax^{b}+c$	T	0.9922	0.1049
	BMI	0.9957	0.0777
	T*BMI	0.9921	0.1053
	W/H	0.9971	0.0643

**Table 3:** Demographic characteristics of the 14 randomly selected subjects. Data are expressed as mean ± standard deviation.

Age (Years)	Height (cm)	Weight (kg)	BMI (kg/m^2^)	Thickness (cm)	Exposure Index
30.8 ± 11.9	174.1 ± 5.3	78.5 ± 14.9	28.9 ± 4.7	20.3 ± 4.5	151.9 ± 23.6

## Data Availability

Not applicable.
